# Low‐dose fluconazole as a useful and safe prophylactic option in patients receiving allogeneic hematopoietic stem cell transplantation

**DOI:** 10.1002/cam4.6815

**Published:** 2024-01-11

**Authors:** Kentaro Hirade, Shigeru Kusumoto, Hiroya Hashimoto, Kazuhide Shiraga, Shinya Hagiwara, Kana Oiwa, Tomotaka Suzuki, Shiori Kinoshita, Masaki Ri, Hirokazu Komatsu, Shinsuke Iida

**Affiliations:** ^1^ Department of Hematology and Oncology Nagoya City University Institute of Medical and Pharmaceutical Sciences Nagoya Japan; ^2^ Department of Hematology and Cell Therapy Aichi Cancer Center Hospital Nagoya Japan; ^3^ Clinical Research Management Center of Nagoya City University Hospital Nagoya Japan

**Keywords:** allogeneic hematopoietic stem cell transplantation, fluconazole, fungal infection, fungal prophylaxis

## Abstract

**Background:**

Invasive fungal infections (IFIs) represent a potentially fatal complication in patients who undergo allogeneic hematopoietic stem cell transplantation (HSCT) if the initiation of therapy is delayed. Some guidelines recommend antifungal prophylaxis or preemptive therapy for these patients depending on the risk of IFIs following allogeneic HSCT. This retrospective study aimed to identify the group of patients who safely undergo allogeneic HSCT with low‐dose fluconazole (FLCZ) prophylaxis (100 mg/day).

**Methods:**

We retrospectively reviewed 107 patients who underwent their first allogeneic HSCT at Nagoya City University Hospital from January 1, 2010, to December 31, 2019. We analyzed the efficacy of low‐dose FLCZ prophylaxis and investigated the relationship between major risk factors and antifungal prophylaxis failure (APF) within 100 days post‐transplant.

**Results:**

Of the 107 patients, 70 received low‐dose FLCZ prophylaxis, showing a cumulative incidence of APF of 37.1% and a proven/probable IFI rate of 4.3%. There were no fungal infection‐related deaths, including *Aspergillus* infections, in the FLCZ prophylaxis group. In a multivariable analysis, cord blood transplantation (CBT) (subdistribution hazard ratio (SHR), 3.55; 95% confidence interval (CI), 1.44–8.77; *p* = 0.006) and abnormal findings on lung CT before transplantation (SHR, 2.24; 95% CI, 1.02–4.92; *p* = 0.044) were independent risk factors for APF in the FLCZ prophylaxis group.

**Conclusion:**

Low‐dose FLCZ prophylaxis is a useful and safe option for patients receiving allogeneic HSCT, except in those undergoing CBT or having any fungal risk features including history of fungal infections, positive fungal markers, and abnormal findings on lung CT before transplantation.

## 1 INTRODUCTION

The mortality rate of invasive fungal infections (IFIs) in hematopoietic stem cell transplantation (HSCT) remains high.^1^ Therefore, appropriate prevention and early diagnosis and treatment of IFIs are important.^2^, ^3^ Fluconazole (FLCZ) is the most commonly used antifungal agent for prophylaxis in HSCT,^4^ and it significantly reduces the incidence of IFI and all‐cause mortality.^5^ According to the recent prospective study in China, FLCZ 64.3%, itraconazole (ITCZ) 15.4%, and voriconazole (VRCZ) 10.6% were selected as single agent prophylaxis of IFIs in 818 allogeneic HSCT from January 2011 to October 2011.^6^ However, FLCZ does not cover *Aspergillus* species,^7^, ^8^ the major causative species of IFIs. Hence, the risk of mold infection must be considered when selecting a prophylactic agent.^9^

Posaconazole (PSCZ) has recently emerged as a new fungal prophylaxis option with a spectrum that covers *Aspergillus*. PSCZ is a novel azole agent that showed both a non‐inferior efficacy to VRCZ in the treatment of invasive aspergillosis and less toxicity.^10^ PSCZ can also be used to treat mucormycosis.^11^ Despite the advent of such novel agents, meta‐analyses have shown that prophylaxis using azoles with anti‐mold activity reduces the cumulative incidence of IFIs but does not reduce all‐cause mortality,^12^ suggesting that it is necessary to identify which patient groups need anti‐mold prophylaxis at pretransplant screening. In addition, there are concerns regarding CYP3A4‐mediated drug interactions, induction of antifungal resistance, and increased medical costs when using these new azole agents.^12^, ^13^, ^14^, ^15^, ^16^ Therefore, FLCZ could be a feasible option because of its weaker CYP3A4 inhibitory effect compared to second‐generation azoles and cost‐effectiveness. The efficacy of low‐dose FLCZ prophylaxis in human leukocyte antigen (HLA)‐matched allografts has been investigated in a recent study,^17^ which showed that the incidence of proven/probable IFI was 5.5%, with only 0.9% of these caused by *Candida non‐albicans* strain. This indicates that low‐dose FLCZ prophylaxis is effective in preventing fungal infections in low‐risk HLA‐matched allogeneic HSCT.

Our retrospective study evaluated the efficacy of low‐dose FLCZ fungal prophylaxis during the first 100 days after allogeneic HSCT to identify the group of patients who genuinely require anti‐mold prophylaxis and can safely receive low‐dose FLCZ prophylaxis without breakthrough infections.

## 2 METHODS

### 2.1 Patients

A total of 107 patients who underwent their first allogeneic HSCT at Nagoya City University Hospital from January 1, 2010, to December 31, 2019 were reviewed. Second transplants performed within 100 days due to engraftment failure were counted as one transplant. Follow‐up ended at the time of the most recent hospital visit or death, and we collected data until March 31, 2021.

### 2.2 Definition of antifungal prophylaxis and antifungal prophylaxis failure (APF)

Antifungal prophylaxis was defined as the use of antifungal agents other than for therapeutic purposes from Day −7 to Day 0 (transplant day). APF was defined as the use of antifungal agents from Day 1 to Day 100 for therapeutic purposes and not for prophylaxis. APF did not account for any changes in antifungal agents due to non‐infectious patient‐related factors such as adverse events, poor oral intake, or cost considerations. APF was classified according to the 2019 EORTIC/MSG diagnostic criteria.^18^ All CT images were evaluated by the attending physicians and radiologists.

### 2.3 Antimicrobial prophylaxis and treatment

All patients were assigned to clean rooms with HEPA filters from the start of the conditioning regimens until at least neutrophil recovery (>0.5 × 10^^9^/L). Physicians selected FLCZ 100 mg/day, VRCZ loading 600 mg/day (maintenance 400 mg/day), micafungin (MCFG) 50 mg/day, or ITCZ 200–400 mg/day for fungal prophylaxis. The choice of the fungal prophylactic agent was made at the attending physician's discretion based on factors such as past fungal infection history, screening lung CT findings, and risk of filamentous fungal infection, and these agents were continued until at least 100 days posttransplant in principle. All patients received antibacterial and antiviral prophylaxis, initiated during the conditioning regimens, with acyclovir 600 mg/day (100 mg/day from day 36), levofloxacin 500 mg/day or ciprofloxacin 600 mg/day, sulfamethoxazole + trimethoprim 400 mg + 80 mg/day, or pentamidine isethionate inhalation 300 mg/month. For these medications, dose reduction, withdrawal, or switching to intravenous formulations were allowed according to the hepatic/renal function, drug interactions, and patient status. In addition, letermovir 480 mg/day (240 mg/day only with cyclosporine [CSP]) has been used for patients seropositive for *cytomegalovirus* (CMV) IgG antibody since 2019.

In cases where clinical symptoms and/or laboratory findings suggested infection, infectious examinations including blood tests, endoscopy including bronchoscopy, CT/MRI imaging, and various culture tests were performed. In principle, when infections caused by fungal pathogens resistant to FLCZ were suspected, a broad‐spectrum antifungal agent was administered, and de‐escalation was performed either when the cause of infection was confirmed as non‐fungal or when improvement was achieved with the treatment.

### 2.4 Graft‐versus‐host disease (GVHD) prophylaxis/treatment

All patients were administered tacrolimus (TAC) or CSP for GVHD prophylaxis. Depending on the stem cell source, HLA status, or disease condition of each case, short‐term methotrexate (MTX) or mycophenolate mofetil (MMF) was also added. There were no cases of anti‐thymocyte globulin (ATG) prophylaxis in our study cohort.

### 2.5 Conditioning regimen

Myeloablative conditioning (MAC) or reduced‐intensity conditioning (RIC) regimens were used. The MAC regimens were busulfan (BU)/cyclophosphamide (CY), cytarabine (AraC)/CY/total body irradiation (TBI), and CY/TBI. The RIC regimens were fludarabine (FLU)/BU/TBI, FLU/BU, FLU/melphalan (MEL), and FLU/MEL/TBI.

### 2.6 Statistical analysis

Comparisons between the FLCZ prophylaxis group and the non‐FLCZ prophylaxis group were analyzed using the Mann–Whitney *U* test for age and Fisher's exact test for the other parameters. The cumulative incidence of post‐FLCZ APF until day 100 was analyzed using Gray's test, and death due to post‐non‐FLCZ APF until day 100 was considered the competing event. The Kaplan–Meier method was used to analyze overall survival (OS). To confirm a significant association between major risk factors and post‐FLCZ APF, a Fine‐Gray regression analysis was performed to calculate the subdistribution hazard ratio (SHR), 95% confidence intervals (CIs), and P values. For the multivariable analysis, those with *p* < 0.2 in the univariate analysis were analyzed. To improve the accuracy of the analysis, “HLA full‐matched” and “Disease condition at conditioning regimen” were not included as risk factors because these were considered to be included in “CBT,” thereby reducing the number of factors analyzed. A two‐sided *p* < 0.05 was considered statistically significant.

## 3 RESULTS

**FIGURE 1:**
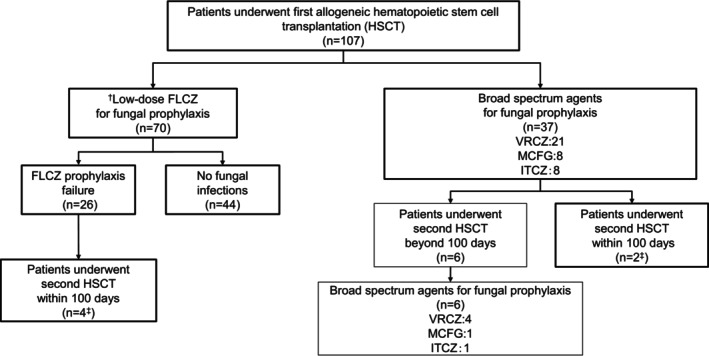
Flowchart of antifungal prophylaxis in the 107 patients who underwent allogeneic HSCT. FLCZ, fluconazole; HSCT, hematopoietic stem cell transplantation; ITCZ, itraconazole; MCFG, micafungin; VRCZ, voriconazole; ^†^Fluconazole (FLCZ): 100 mg/day ^‡^Six patients who underwent their second HSCT within 100 days were considered one transplantation case.

A total of 113 HSCTs were performed on the 107 patients in this retrospective cohort study; of these, 107 were first transplants, and six were second transplants (Figure 1). FLCZ was the most frequently selected agent for fungal prophylaxis at first transplantation (*n* = 70), followed by VRCZ (*n* = 21), MCFG (*n* = 8), and ITCZ (*n* = 8). None of the second HSCT patients received FLCZ prophylaxis.

We compared the clinical characteristics of the 107 patients in the FLCZ prophylaxis and non‐FLCZ prophylaxis groups who underwent their first allogeneic HSCT (Table 1; Tables S1 and S2). FLCZ prophylaxis was more frequently selected than non‐FLCZ prophylaxis in patients with lymphoid malignancy/benign hematological diseases, no history of previous fungal infections, no abnormalities on lung CT before HSCT, and negative β‐D glucan and/or galactomannan antigen.

**TABLE 1:** Baseline characteristics of patients in the FLCZ prophylaxis group/non‐FLCZ prophylaxis group who underwent their first allogeneic hematopoietic stem cell transplantation.

Patients with first HSCTs (*n* = 107)		FLCZ prophylaxis (*n* = 70)	Non‐FLCZ prophylaxis (*n* = 37)	*p*‐Value
Age	Median (range)	47.5 (15–67)	52.0 (19–68)	0.076
Sex	Men/Women	40/30	22 / 15	0.840
Disease types	Myeloid	20	20	0.019
	Lymphoid	46	17	
	Other (Benign)	4	0	
Source of transplantation	Related	17	10	0.620
	Non‐related	47	26	
	Cord	6	1	
Conditioning regimen	MAC	36	13	0.153
	RIC	34	24	
HLA full‐matched	Positive	44	20	0.412
Disease status at transplant	Active	5	4	0.716
History of significant CMV infection	Positive	10	9	0.287
Past history of fungal infections	Positive	6	20	<0.001
Abnormalities in lung CT images	Positive	16	27	<0.001
Fungal serological marker	Positive	0	2	0.117
Second HSCT within 100 days	Yes	4	1	0.657

Abbreviations: CMV, cytomegalovirus; FLCZ, fluconazole; HLA, human leukocyte antigen; HSCT, hematopoietic stem cell transplantation; MAC, myeloablative conditioning; RIC, reduced‐intensity conditioning.

**FIGURE 2:**
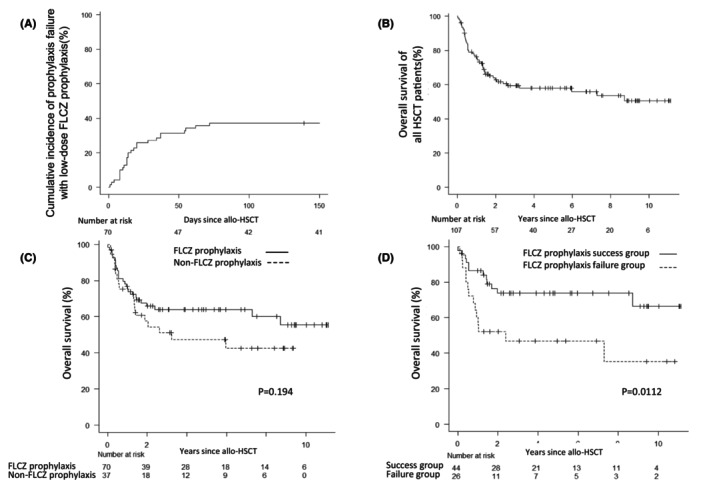
(A) Cumulative incidence of antifungal prophylaxis failure with FLCZ prophylaxis. (B) Overall survival following the first allogeneic HSCT. (C) Overall survival in the FLCZ prophylaxis and non‐FLCZ prophylaxis groups. (D) Overall survival in the success and failure groups of FLCZ prophylaxis. FLCZ, fluconazole; HSCT, hematopoietic stem cell transplantation; allo‐HSCT, allogeneic HSCT.

Among the 70 patients who received FLCZ prophylaxis, 26 experienced APF, with a cumulative incidence of 37.1% (Figure 2A). In the contrast, among of the 37 patients with non‐FLCZ group, 13 APF events were admitted (35.1%). The median duration from HSCT to APF was 14 days (range: 1–72). APFs classified as proven, probable, and possible represented 0% (*n* = 0), 12% (*n* = 3), and 19% (*n* = 5) of all APFs, respectively, and the remaining 18 (70%) were unclassified, partly due to early preemptive therapy when fungal infection was suspected. The most commonly used agent for salvage antifungal therapy for post‐FLCZ APF was liposomal amphotericin B (*n* = 11) (Figure S1), and there were no fungal infection‐related deaths, including *Aspergillus* infections, in the FLCZ prophylaxis group.

Of the 26 patients who experienced post‐FLCZ APF, 11 showed abnormal findings on lung CT at pretransplant screening (Table S3). The majority of these findings were characterized by nodular/ground glass mixed patterns (27.3%), followed by small nodular patterns (18.2%), and ground glass patterns (18.2%). Nine patients were judged by their physicians as having an inactive pulmonary infection. New pulmonary lesions at pretransplant screening were observed in two patients: one was treated for bacterial pneumonia and improved immediately, and the other showed a new 1 mm nodule without any clinical symptoms, which was diagnosed as a non‐infectious lesion.

In the entire study cohort (*n* = 107), the estimated median OS (mOS) was not reached (range: 0–133, Figure 2B). When analyzed separately for the FLCZ and non‐FLCZ prophylaxis groups, the mOS was not reached in the former group and was 38.9 months in the latter group (*p* = 0.194, Figure 2C). Subsequently, the success and failure groups of FLCZ prophylaxis were analyzed. The success group showed a mOS of 41.5 months, whereas the failure group showed a mOS of 13.5 months (*p* = 0.0112, Figure 2D).

In the multivariable analysis, cord blood transplantation (CBT) (SHR, 3.55; 95% CI, 1.44–8.77; *p* = 0.006) and abnormal findings on lung CT before transplant (SHR, 2.24; 95% CI 1.02–4.92; *p* = 0.044) were identified as independent risk factors for post‐FLCZ APF (Table 2).

**TABLE 2:** Analysis of risk factors for failure of antifungal prophylaxis with FLCZ.

		Univariate			Multivariable		
		SHR	95% CI	*p*‐Value	SHR	95% CI	*p*‐Value
Age	≥40 vs <40	1.31	0.54–3.198	0.554	NA	NA	NA
Sex	Men vs. women	0.78	0.37–1.67	0.525	NA	NA	NA
Disease	Myeloid vs. lymphoid, other	1.37	0.63–2.98	0.427	NA	NA	NA
Stem cell source	Related	Ref			Ref		
	Non‐related	0.64	0.26–1.59	0.338	0.83	0.33–2.07	0.687
	Cord	3.62	1.53–8.55	0.003	3.55	1.44–8.77	0.006
HLA full‐matched	Yes vs. no	0.53	0.25–1.12	0.095	NA	NA	NA
Conditioning regimen	MAC vs. RIC	0.94	0.44–1.99	0.865	NA	NA	NA
Disease status at transplant	Active vs. stable	2.38	1.16–4.87	0.017	NA	NA	NA
History of significant CMV infection	Positive vs. negative	1.68	0.63–4.49	0.301	NA	NA	NA
Past fungal infections	Positive vs. negative	1.59	0.48–5.28	0.447	NA	NA	NA
Abnormalities in lung CT images	Positive vs. negative	2.81	1.36–5.78	0.005	2.24	1.02–4.92	0.044

Abbreviations: CI, confidence interval; CMV, cytomegalovirus; FLCZ, fluconazole; MAC, myeloablative conditioning; NA, not applicable; Ref, reference; RIC, reduced‐intensity conditioning; SHR, subdistribution hazard ratio.

## 4 DISCUSSION

This retrospective analysis showed that a proportion of patients safely underwent allogeneic HSCT using low‐dose FLCZ prophylaxis for 100 days post‐transplant. In our study, the cumulative incidence of APF was 37.1%, but the proven/possible IFI rate was 4.3%, and there were no fungal‐related deaths in the FLCZ prophylaxis group. Importantly, a multivariable analysis demonstrated that CBT and abnormal findings on lung CT at pretransplant screening were independent risk factors for post‐FLCZ APF. This result represents a new finding that further stratifies the risk of mold‐fungal infection before HSCT with FLCZ prophylaxis.

Fungal prophylaxis using agents with anti‐mold activity, such as VRCZ and PSCZ, has been widely adopted in high‐risk populations, such as in patients with acute myeloid leukemia or myelodysplastic syndrome and those undergoing HSCT, to reduce the IFI risk and improve patient outcomes.^19^, ^20^ PSCZ is a new broad‐spectrum azole with anti‐*Aspergillus* and anti‐*Mucor* activities. Comparative studies have shown a reduction in the incidence of IFI with FLCZ and ITCZ in neutropenic patients^21^ and with FLCZ in patients with severe GVHD.^22^ However, problems with the prophylactic use of PSCZ include its high cost and strong CYP3A4 inhibitory effect, which has a significant impact on key drugs for GVHD prevention, such as CSP and TAC, which are frequently used in transplantations.^23^ Despite its broader spectrum, there are concerns regarding the presence of breakthrough infections, the unknown primary therapy after breakthrough, and the possibility of inducing highly resistant strains due to selection pressure.^15^, ^16^, ^24^, ^25^, ^26^ Antifungal drug‐resistant pathogens are on the rise worldwide and pose new threats to patient management and clinical success.^27^

FLCZ is a first‐generation triazole antifungal agent that primarily targets *C. albicans* and is characterized by weaker CYP3A4 inhibition than second‐generation azoles and cost‐effectiveness; however, it lacks anti‐*Aspergillus*/*Mucor* activity.^28^ In Japan, a dose of 100–200 mg/day is often chosen for FLCZ prophylaxis,^29^ whereas international guidelines recommend 400 mg/day, which is based on the report by Goodman et al.^30^ They stated that this dose was selected because of the highly immunosuppressed status of the patient and the expectation of *Aspergillus* coverage, and the decision was not based on scientific evidence. FLCZ prophylaxis in allogeneic HSCT has been reported to induce a reduction in intestinal mucosal destruction by decreasing intestinal *Candida* colony formation and the incidence of intestinal GVHD, thereby reducing mortality.^31^ Regarding the prophylactic effect of doses less than 400 mg of FLCZ, it has been reported that the inhibitory effect of these doses on colony formation and IFI incidence is not inferior to that of the 400 mg dose in autologous and allogeneic HSCT.^32^, ^33^ The cumulative incidence of IFI (proven/probable/possible) in our study was 11.4%, similar to the 11.7% reported in previous studies showing the efficacy of a 100 mg dose in HLA‐matched allograft HSCT.^17^

Previous studies have reported an 8.4% cumulative incidence of proven invasive candidiasis with low‐dose FLCZ prophylaxis in HLA‐mismatched bone marrow transplantation,^34^ but no cases of invasive candidiasis were observed in the current study. It is also noteworthy that there were no IFI‐related mortality events in our study. These results suggest that even if a breakthrough infection occurs with FLCZ, the infection may not be fatal given appropriate early interventions such as preemptive broad‐spectrum antifungal therapy, removal of central venous catheters, and other infectious source control. History of active leukemia, CBT, and fungal infections have previously been reported as pre‐engraftment IFI risk factors,^35^ whereas delayed engraftment, high‐dose corticosteroid use for >1 week, iron overload, CMV infection, and acute GVHD have been reported as post‐engraftment IFI risk factors.^36^ In addition to CBT, our analysis identified abnormal findings on lung CT before HSCT as an independent risk factor associated with post‐FLCZ APF.

High galactomannan antigen levels indicate invasive *Aspergillus* infections and mortality in neutropenic patients.^37^, ^38^ Therefore, we did not choose FLCZ for cases positive for galactomannan antigen. In a previous representative comparative study between PSCZ and FLCZ for patients with GVHD, no difference in the incidence of IFIs was found in patients who were negative for galactomannan antigen in the majority of cases at baseline.^22^ This result implies that some of the patients administered antifungal prophylaxis agents with anti‐mold activity, such as VRCZ or PSCZ, do not require broad‐spectrum prophylaxis.

Our study has some limitations. First, this study was a retrospective analysis of a single institution in which antifungal drugs for prophylaxis or preemptive therapy were selected at the attending physician's discretion. Second, the APFs in our study were often diagnosed as “unclassified (70%),” which may have resulted in a low estimate of the proven/probable IFI rate (4%). However, this was because we provided early therapeutic intervention when a fungal infection was suspected. Third, APF did not account for changes in antifungal agents due to adverse events, cost, or poor oral intake. Finally, there were regional specificities such as fungal distribution and susceptibility. Therefore, instead of interpreting these data as is, we need to consider an antifungal prophylaxis strategy according to the characteristics of each region.^39^

## 5 CONCLUSION

This study suggests that low‐dose FLCZ is a useful and safe prophylactic option for 100 days posttransplant in patients receiving allogeneic HSCT, except in those undergoing CBT or having any fungal risk features including history of fungal infections, positive fungal markers, and abnormal findings on lung CT at pretransplant screening. Well‐designed clinical studies are warranted to establish appropriate individualized antifungal prophylaxis strategies in allogeneic HSCT in the era of novel azole agents.

## AUTHOR CONTRIBUTIONS

**Kentaro Hirade:** Conceptualization (equal); data curation (lead); formal analysis (lead); investigation (lead); methodology (equal); project administration (equal); software (equal); visualization (lead); writing – original draft (lead). **Shigeru Kusumoto:** Conceptualization (equal); formal analysis (supporting); investigation (supporting); methodology (supporting); project administration (equal); software (supporting); supervision (equal); validation (supporting); visualization (supporting); writing – review and editing (lead). **Hiroya Hashimoto:** Formal analysis (equal); methodology (supporting); software (equal); supervision (equal); visualization (equal); writing – review and editing (supporting). **Kazuhide Shiraga:** Writing – review and editing (supporting). **Shinya Hagiwara:** Writing – review and editing (supporting). **Kana Oiwa:** Writing – review and editing (supporting). **Tomotaka Suzuki:** Writing – review and editing (supporting). **Shiori Kinoshita:** Writing – review and editing (supporting). **Masaki Ri:** Writing – review and editing (supporting). **Hirokazu Komatsu:** Writing – review and editing (supporting). **Shinsuke Iida:** Project administration (supporting); resources (equal); supervision (equal); writing – review and editing (supporting).

## CONFLICT OF INTEREST STATEMENT

The authors declare that they have no conflict of interest.

## ETHICS STATEMENT

The Institutional Review Board of Nagoya City University Hospital approved this study (No.60‐20‐0104), which conformed to the guidelines of the Declaration of Helsinki, and since any human samples were not used in the retrospective analysis, an exemption from obtaining an informed consent document was granted.

## Supporting information


Figure S1.

Table S1.

Table S2.

Table S3.

## Data Availability

Data are available from the authors upon reasonable request.
