# Supplementary material for: Low‐dose fluconazole as a useful and safe prophylactic option in patients receiving allogeneic hematopoietic stem cell transplantation
Source: Cancer Med. 2024 Jan 11;13(3):e6815. doi: 10.1002/cam4.6815 (PMC10905229; doi:10.1002/cam4.6815)
Supplement: Supplementary file 1 — Figure S1. [file CAM4-13-e6815-s003.pdf]

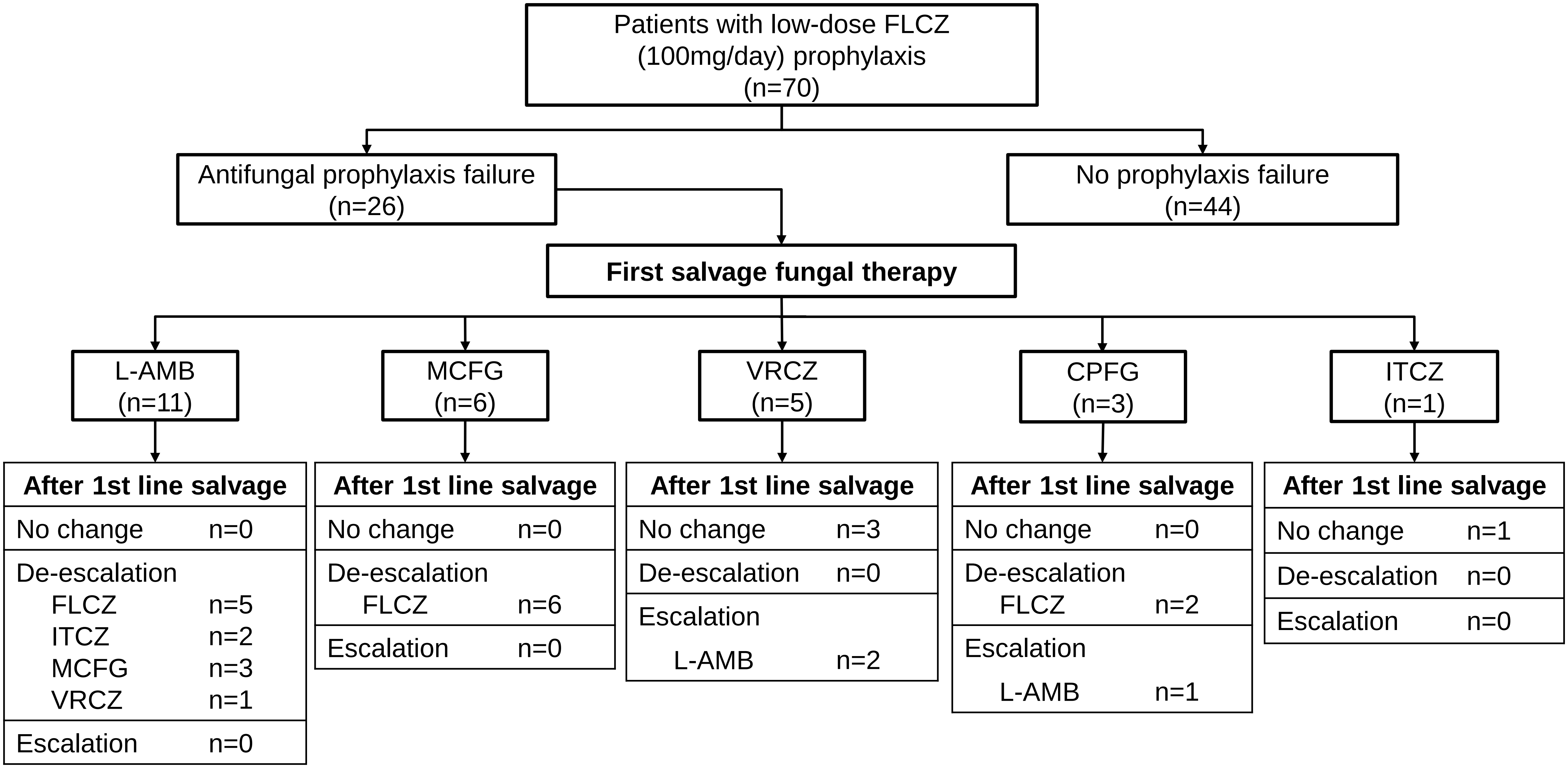

Supplementary Figure 1. Flowchart of fungal outcomes with FLCZ prophylaxis.

Abbreviations: FLCZ, fluconazole; MCFG, micafungin; VRCZ, voriconazole; ITCZ, itraconazole, amphotericin B; L-AMB, caspofungin; CPFG
