# Supplementary material for: Low‐dose fluconazole as a useful and safe prophylactic option in patients receiving allogeneic hematopoietic stem cell transplantation
Source: Cancer Med. 2024 Jan 11;13(3):e6815. doi: 10.1002/cam4.6815 (PMC10905229; doi:10.1002/cam4.6815)
Supplement: Supplementary file 2 — Table S1. [file CAM4-13-e6815-s004.pdf]

Supplementary Table 1. Disease types in the FLCZ prophylaxis and non-FLCZ prophylaxis groups

Disease types		Total number of first HSCT (n=107)	FLCZ prophylaxis (n=70)	Non-FLCZ prophylaxis (n=37)
Myeloid	AML	32	16	16
	MDS/CMMoL	5	2	3
	BAL(Myeloid)	3	2	1
	CML BC (Myeloid)	1	1	0
Lymphoid	Ph+ALL	3	3	0
	Ph-ALL	6	5	1
	BAL(Lymphoid)	2	2	0
	CML BC(Lymphoid)	4	2	2
	DLBCL	9	8	1
	FL	2	1	1
	PTCL	5	5	0
	ATLL	23	12	11
	TLBL	2	1	1
	NK/T Lymphoma	5	5	0
	MM	1	1	0
	AA	4	4	0

HSCT, hematopoietic stem cell transplantation; FLCZ, fluconazole; AML, acute myeloid leukemia; MDS, myelodysplastic syndrome; CMMoL, chronic myelomonocytic leukemia; BAL, biphenotypic acute leukemia; CML BC, chronic myeloid leukemia blastic crisis; Ph, Philadelphia; ALL, acute lymphoblastic leukemia; DLBCL, diffuse large b cell lymphoma; FL, follicular lymphoma; PTCL, peripheral t cell lymphoma; ATLL, adult t cell leukemia/lymphoma; TLBL, t lymphoblastic leukemia/lymphoma; MM, multiple myeloma; AA, aplastic anemia
