# Supplementary material for: Low‐dose fluconazole as a useful and safe prophylactic option in patients receiving allogeneic hematopoietic stem cell transplantation
Source: Cancer Med. 2024 Jan 11;13(3):e6815. doi: 10.1002/cam4.6815 (PMC10905229; doi:10.1002/cam4.6815)
Supplement: Supplementary file 3 — Table S2. [file CAM4-13-e6815-s001.pdf]

Supplementary Table 2. Donor sources, number of HLA-matched alleles, and conditioning regimens in the FLCZ and non-FLCZ prophylaxis groups

	Donor source	Total number of first HSCT (n=107)	FLCZ prophylaxis (n=70)	Non-FLCZ prophylaxis (n=37)
Related	Bone marrow	25	16	9
	Peripheral blood	2	1	1
Un-related	Bone marrow	73	47	26
	Peripheral blood	0	0	0
	Cord	7	6	1
	**HLA matched allele**	**Total number of first HSCT (n=107)**	**FLCZ prophylaxis (n=70)**	**Non-FLCZ prophylaxis (n=37)**
Matched	8/8	38	28	10
	6/6(sibling)	26	16	10
Unmatched	7/8	32	18	14
	6/8	4	2	2
	5/8	5	4	1
	4/8	2	2	0
**Intensity**	**Conditioning regimen**	**Total number of first HSCT (n=107)**	**FLCZ prophylaxis (n=70)**	**Non-FLCZ prophylaxis (n=37)**
MAC	BU/CY	44	32	12
	CY/TBI	1	1	0
	AraC/CY/TBI	4	3	1
RIC	FLU/BU	17	8	9
	FLU/BU/TBI	36	22	14
	FLU/MEL	1	1	0
	FLU/MEL/TBI	4	3	1

HSCT, hematopoietic stem cell transplantation; HLA, human leukocyte antigen; MAC, myeloablative conditioning; RIC, reduced intensity conditioning;
BU; busulfan, CY; cyclophosphamide, AraC, cytarabine; TBI, total body irradiation; FLU, fludarabine; MEL, melphalan
