# Supplementary material for: Low‐dose fluconazole as a useful and safe prophylactic option in patients receiving allogeneic hematopoietic stem cell transplantation
Source: Cancer Med. 2024 Jan 11;13(3):e6815. doi: 10.1002/cam4.6815 (PMC10905229; doi:10.1002/cam4.6815)
Supplement: Supplementary file 4 — Table S3. [file CAM4-13-e6815-s002.pdf]

Supplementary Table 3. Detailed information on abnormal lung CT findings at pre-transplant screening in the 11 patients with FLCZ prophylaxis failure

Case	Abnormality findings' patterns in screening CT before HSCT	"Obsolete" † or not	MAX size	Numbers of lesion
1	GGO	Obsolete	12.3mm	×2
2	Nodule	Obsolete and new	23mm	×3
3	GGO/Nodule	Obsolete	10.7mm	×2/×1
4	Nodule/GGO	Obsolete	9.7mm	×1/×2
5	Nodule /Linear lesion	Obsolete	17.4mm	×1/×1
6	Nodule	Obsolete	7.5mm	×1
7	GGN	New	2.6mm	×1
8	GGO	Obsolete	3.0mm	×1
9	Nodule/GGO	Obsolete	7.0mm	×3
10	Granular	Obsolete	1.0mm	×1
11	Nodule	Obsolete	4.0mm	×1

†"Obsolete" means no change of abnormal findings among at least two times of CT examinations.
HSCT, hematopoietic stem cell transplantation; GGO, Ground-grass opacity; GGN: Ground-grass nodule
